# The role of spermidine in plants and humans: a pathway from climate change adaptation to health benefits

**DOI:** 10.1038/s41538-025-00695-2

**Published:** 2026-02-09

**Authors:** Bojana D. Blagojević, Sophie Brunel-Muguet, Rada Šućur, Velimir Mladenov, Igor Balaž, Johann Vollmann, Vasileios Fotopoulos, Karsten Mäder

**Affiliations:** 1 University of Novi Sad, Faculty of Agriculture, Trg Dositeja Obradovića 8, 21000 Novi Sad, Serbia; 2 From Farm to Pharm Group, Novi Sad, Serbia; 3 Normandie Université, UNICAEN, INRAE, UMR 950 Ecophysiologie Végétale, Agronomie et Nutritions N, C, S, Caen, France; 4 PanCrop Lab, Novi Sad, Serbia; 5 Department of Agricultural Sciences, BOKU University, Tulln an der Donau, Austria; 6 Department of Agricultural Sciences, Biotechnology and Food Science, Cyprus University of Technology, 3603 Lemesos, Cyprus; 7 Institute of Pharmacy, Martin Luther University Halle-Wittenberg, Kurt-Mothes-Str. 3, 06120, Halle (Saale), Germany

**Keywords:** Plant sciences, Biochemistry, Metabolomics

## Abstract

Growing demands for healthier diets are driving agricultural and food scientists to develop climate-resilient crops and food systems that ensure nutritionally effective food. Beyond providing basic energy requirements, nutrients may actively influence human physiology and health. One such molecule, spermidine, a polyamine abundant in wheat and soybean, has attracted particular interest. From the aspect of human health, spermidine is mainly studied for healthy ageing properties and has been associated with cardioprotective, neuroprotective, and anti-cancerogenic effects. On the other hand, being present in all plants, spermidine is essential for growth, development, and stress adaptation. Endogenously or when exogenously applied, spermidine can help plants adapt to harsh climate change conditions. Bringing together current knowledge on the significance of spermidine in both plants and humans, this review aims to trace its journey *From Farm to Pharm*, highlighting its importance for sustainable crop production, improved nutrition, and emerging pharmacological applications.

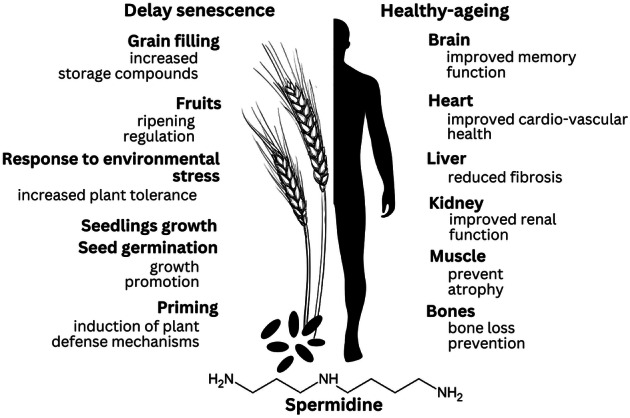

## Introduction

Agriculture is the backbone of human society. From the first settlements, people have been searching for mechanisms to improve crop production and the nutritional values of food. During previous decades, we witnessed a remarkable increase in crop yields. The reason for such an improvement lies mainly in the *Green Revolution*, supported by the mineral fertilizers and genetic achievements of the breeders^1,2^. Although genetics has played a central role in generating more productive varieties, exhibiting increased resistance to diseases and pests, and better adapting to different environmental conditions, agricultural and food systems are now facing serious obstacles, with highly unpredictable environmental factors, non-resilience of food chains, hunger, and malnutrition being the major ones^3^.

Climate change, with extreme temperature changes, floods, drought and consequently, limited access to water for irrigation, poor soil quality, loss of biodiversity, pests and diseases, *etc*., are some of the wide array of obstacles that farmers are facing presently^4–6^. On the other side, despite the achievements and technological advancements in food production, food-related health conditions such as obesity and malnutrition, and noncommunicable chronic diseases such as cardiovascular diseases and diabetes are responsible for more than 74% of all the deaths globally^7^. This is an alarming sign that should encourage our global and local food systems to undertake profound and systemic changes. Therefore, there is an increasing demand and additional pressure on agricultural and food science to provide innovative solutions that will ensure stable and healthy food production^8^.

*From Farm to Pharm* is an illustrative name for our initiative to highlight important aspects of the relationship between agriculture and human health. *From Farm to Pharm* encompasses, but is not limited to: (i) growing crops and climate change adaptation, (ii) *Precision Food* production – food that meets a person’s unique needs, and (iii) agricultural crops as sources of pharmaceuticals. It is forcing a one-health perspective, i.e., that the whole food production processes and developed food products need to be healthy and sustainable for all: humans, animals, plants, the environment, and our planet.

Spermidine perfectly fits the *From Farm to Pharm* pathway. It is ubiquitously present in all eukaryotes, including plants and humans, and plays crucial roles in multiple cellular functions. It belongs to the group of polyamines, which are biogenic amines with more than two amino groups^9^. At physiological pH, spermidine is positively charged and that is regarded as a key characteristic for some of its biological roles. Positive charge enables spermidine interactions with negatively charged molecules, such as DNA, RNA, ribosomes, proteins, phospholipids i.e., cell membranes, thereby stabilising these macromolecular structures^10,11^.

Spermidine is involved in growth and proliferation, organogenesis, transcription and translation, the control of the activity of ion channels, immune response, initiation of autophagy, prevention of senescence, response to oxidative, osmotic, and other stresses, thus being one of the essential metabolites^12–16^.

Wheat germs and soybean seeds are the richest plant-based sources of spermidine, with concentrations of approximately 300 mg and 200 mg of spermidine per kg, respectively^17^. Importantly, spermidine content can significantly vary within the same species, depending on plant genetics and environmental growing conditions^18^.

The most represented biogenic (i.e., biologically formed) aliphatic amines in plants and humans are listed in Table 1. For each polyamine, it is presented its chemical structure and whether it is present in plants or mammals, i.e., humans. Polyamines are classified by their structure and the number of amino groups they contain, which also determines their chemical behaviour and biological roles. The structures are characterised by primary amino groups at each end of the molecule, while triamines and tetraamines also possess secondary amino groups in the backbone^19^.

**Table 1:** Major diamines and polyamines present in plants and humans

Name	Chemical structure**	Plants	Humans
Putrescine		✓	✓
Cadaverine		✓*	✗
Spermidine		✓	✓
Spermine		✓	✓
Thermospermine		✓	✗
Agmatine		✓	✓*

The sign “✓” denotes that a compound is present in a plant or human organism, and the mark “✗” denotes that it is not present. Cadaverine and thermospermine are not present in the human organism, only in plants. *Cadaverine is not found in all plants, only in legumes and some other families^144^. Agmatine is not synthesised in human cells, but it is synthesised by gut bacteria, so it can be found in human organism^12^.

**It should be noted that at physiological pH values, the nitrogen atoms in these structures would be predominantly protonated.

The most examined polyamines are putrescine, spermidine and spermine. Spermidine represents the central polyamine in both biosynthetic and catabolic pathways of these three, as it occupies the intermediate position between putrescine and spermine^20^. Putrescine is the simplest and earliest product in the polyamine biosynthetic pathway, primarily serving as a precursor with more limited and less specific biological effects. Spermidine, synthesized from putrescine by the addition of an aminopropyl group, displays greater physiological specificity and regulates key processes such as translation, membrane stability, and autophagy^21^. Spermine results from the further conversion of spermidine and generally accumulates at lower levels. Although it is important for nucleic acid stabilization and ROS scavenging, its biological roles are narrower and more context-dependent^22^. Thus, while all three polyamines contribute to cellular function, spermidine stands out due to its unique combination of abundance, broad physiological relevance, and strong mechanistic links to health and stress resilience in both plants and humans.

Furthermore, in plants, putrescine is an important metabolite involved in development and resistance to stress^23^, but in humans, it is mainly important as a precursor of spermidine and spermine that are more engaged in health and disease processes. Although the majority of published literature uses the term *polyamines* for all of these structures, some authors, focused to research of health effects of spermidine, suggest the distinction in terms should be made, using the term diamine for putrescine and the term polyamines for spermidine and spermine, highlighting their different biological roles regarding health-promoting effects in humans and other mammals^9^.

## Modes of action of spermidine at the molecular level

Despite being involved in multiple physiological processes, the molecular mechanisms of action are still not completely clear and defined for spermidine. Here, we provide an overview of some of the best-described mechanisms, such as hypusination, autophagy, and pathways linked to oxidative stress.

Spermidine plays a crucial role in both plants and humans by enabling the activation of the translation factor eIF5A through a unique post-translational modification called **hypusination**. eIF5A is a highly conserved, essential protein found in all eukaryotes and archaea, and the fact that hypusination occurs exclusively on this single protein highlights its fundamental biological importance^24^. While bacteria possess a related factor (EF-P), they rely on a different activation mechanism that does not use spermidine^25^. Hypusination happens in two steps: first, deoxyhypusine synthase (DHS) transfers part of the spermidine molecule to a specific lysine on eIF5A, and then deoxyhypusine hydroxylase (DOHH) completes the modification to produce the active form. Activated eIF5A supports protein synthesis, particularly helping ribosomes translate proline-rich sequences that otherwise cause stalling, and its loss leads to growth arrest or defective cell differentiation^26,27^. These findings underscore how essential both spermidine and the hypusination pathway are for normal cellular function.

One of the most pronounced roles of spermidine in humans is being the longevity elixir, by promoting the **autophagy** – a homoeostatic, intracellular degradation and recycling mechanism^16^. Spermidine was found to induce autophagy in cultured yeast and mammalian cells, as well as in nematodes and flies by several mechanisms^28^. In the nucleus, spermidine inhibits histone acetyl transferases (HATs), such as Iki3p and Sas3p, thereby reducing the acetylation of histone H3, and perhaps of other proteins. This leads to epigenetic reprogramming of the transcriptome, resulting in an enhanced expression of autophagy-related genes in the context of a general silencing of many other genes^29^. In cytoplasm, rapid autophagy induction by spermidine administration is achieved through inhibition of the lysine acetyltransferase EP300 (E1A-binding protein p300), primarily resulting in autophagy-relevant cytosolic protein deacetylation^30^.

Emerging evidence suggests the possible induction of autophagy in plants. Polyamines, including spermine and spermidine, can trigger autophagy in plant cells via nitric oxide (NO)/ reactive oxygen species (ROS) signalling and likely share elements with the eukaryotic autophagy machinery, though the mechanistic details remain less worked-out than in animals^31^.

In both plant and human cells, polyamines play dual roles in **maintaining cellular oxidative homoeostasis** by both protecting against free radical–mediated damage and acting as substrates for enzymes that produce reactive oxygen species (ROS)^16^. Oxidative stress occurs when ROS, such as those derived from hydrogen peroxide (H_2_O_2_), exceed the physiological levels required for normal redox reactions and cell signalling. The resulting oxidative damage to macromolecules is associated with senescence, ageing, and a variety of pathological conditions^32^. Polyamines, including spermidine, are found to be potent scavengers of hydroxyl radical and spermine or spermidine can also quench singlet oxygen at higher concentrations^33^. Polyamines efficiently scavenge alkyl (carbon-centred, R•) and peroxy (oxygen-centred, ROO•) radicals, ensuring protection of easily oxidizable compounds, like polyunsaturated fatty acids, in biological systems^34^. On the contrary, enzymatic degradation of polyamines produces ROS. Polyamine oxidases (PAOs) either terminally degrade polyamines or catalyse their back-conversion, transforming spermine into spermidine and spermidine into putrescine. Both the catabolic and back-conversion pathways generate H_2_O_2_ as a by-product^32^. H_2_O_2_ and other ROS that act as signalling molecules and interact with nitric oxide trigger downstream stress-response pathways, stomatal and ion flux regulation, and defence gene expression in plants. Controlled PA oxidation is therefore a signalling hub for adaptation to abiotic/biotic stress^35^.

In plants, polyamines also play crucial roles by cross-talk with hormones and metabolic pathways. Polyamines interact with phytohormone signalling (ABA, auxin), calcium signalling and metabolic networks; exogenous or endogenous spermidine can enhance photosynthetic capacity, osmotic adjustment and transcriptional programmes that increase tolerance to drought, salinity and temperature extremes^36^.

## Roles of spermidine in plants: Plant development, potential to confront environmental stresses, and nutrient management

### Endogenous roles of spermidine

Spermidine has multiple roles in the regulation of various developmental processes in plants from embryogenesis through organogenesis, flowering, and fruit development and maturation^19,37,38^. Plants produce spermidine in various organs at different levels. Research showed that spermidine is the least abundant polyamine in leaves, but is mainly found in all the other organs^39^. This organ-specific distribution pattern reflects its timely roles throughout the plant growth cycle^38^.

Over embryogenesis, because of their polycation characteristics (i.e., ability to bind negatively charged molecules through ionic and hydrogen bonds), polyamines contribute to the acquisition of the zygote polarity, the differentiation of cell layers, and eventually meristem formation. More specifically, spermidine biosynthesis was shown to increase during somatic embryogenesis in *Citrus sinensis*^40^. Similarly, it was demonstrated that increased levels of spermidine were mainly observed at advanced developmental stages (torpedo, pre-cotyledonary, and cotyledonary-staged somatic embryos) in *A. sellowiana*, in contrast to putrescine that was also observed at the globular and heart stages^41^. From a mechanistic perspective, it was shown that spermidine was required for the posttranslational activation of the eukaryotic translation initiation factor eIF5A (i.e., hypusination), essential for cell division^42,43^.

The role of spermidine has also been highlighted in pollen development^42^. This function relies on polyamine homoeostasis and, in particular, on polyamine conjugation, as it was shown that hydroxycinnamoyl-spermidine conjugates act on pollen tube growth^44^. More precisely, the release of H_2_O_2_ from spermidine oxidation mediates pollen tube growth^45^.

Eventually, spermidine was shown to impact later growth and development stages. For example, studies in cereals demonstrated the effects of endogenously synthesised or exogenously supplied spermidine on the quantity or quality of grain storage compounds. It was shown that the direct synthetic pathway from putrescine to spermidine in the grain is a key factor in promoting grain filling and thousand-grain weight in wheat^46^. More about this will be addressed in section 2.2.

An increase in spermidine and other free polyamines has also been associated with fruit maturation-related processes^47^. In tomato, high levels of spermidine and spermine were shown to boost several metabolic processes, such as amino acids and sugar syntheses, and to control the accumulation of various ripening compounds, therefore contributing to the regulation of ripening rate^48,49^. Differences among polyamines were observed, thus raising the fine-tuning of polyamine homoeostasis in the induction of physiological processes. While spermidine and spermine contents prompted sugar, ethylene, and lycopene changes to induce ripening, putrescine had opposite effects^50^. In strawberries, fruit coloration was correlated to high levels of spermidine along with the contribution of abscisic acid (ABA), indole-3-acetic acid (IAA), and ethylene in a coordinated manner^51^.

Overall, the extent of the involvement of spermidine in developmental processes is not only counterbalanced by the regulation of polyamine homoeostasis but also by the interactions with various hormones and precursors of key physiological and biochemical processes throughout the whole plant life.

### Exogenously applied spermidine

**Fig. 1:**
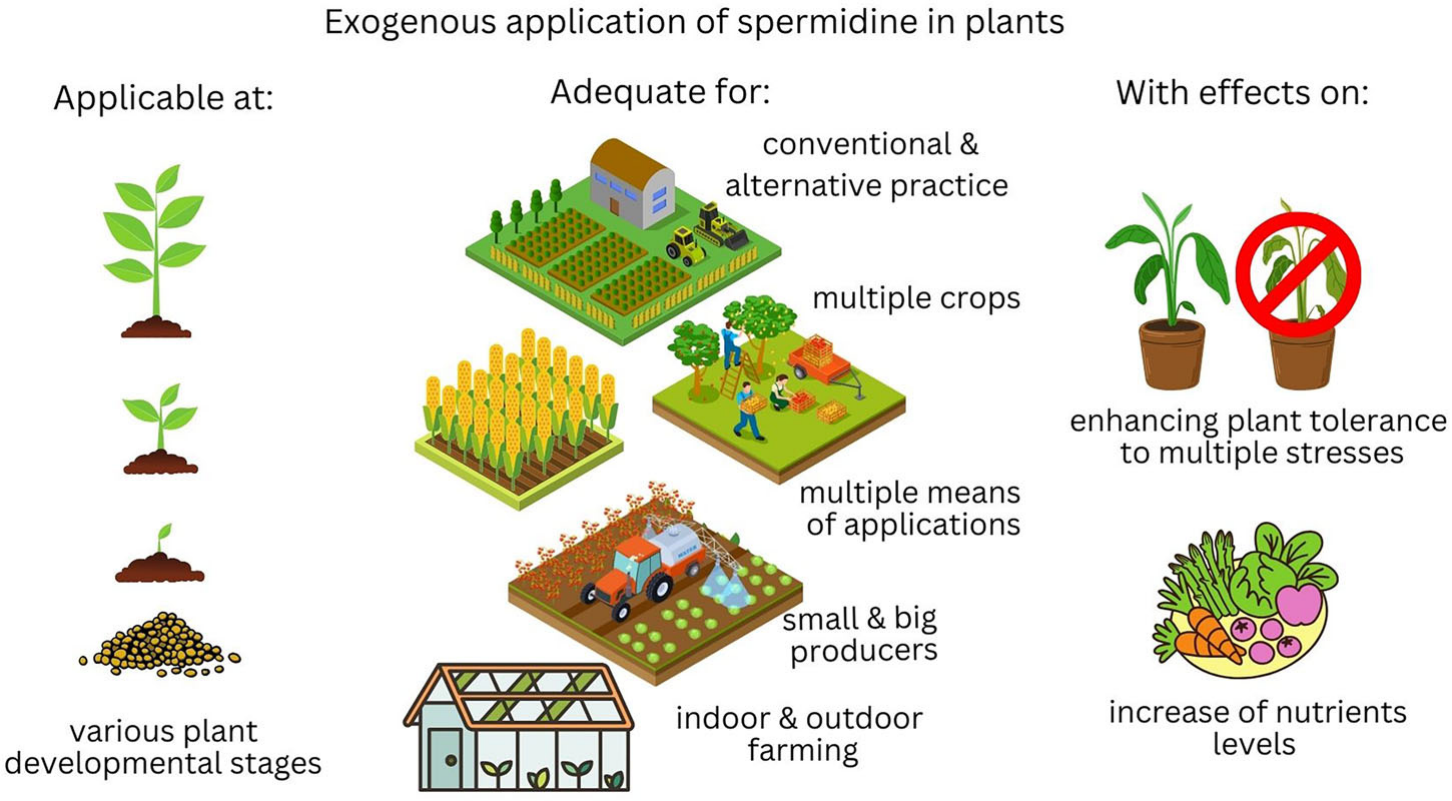
Exogenous applications of spermidine in plants. Plant development stages at which spermidine can be applied (on the left), examples on modes of applications (in the middle), beneficial effects (on the right).(The figure is created by Canva online tool).

Possibly the highest advantages of exogenous applications of spermidine i.e., plant priming, can be observed through the enhancement of plant tolerance to multiple stresses and the increase in nutrient content. Some of the advantages of spermidine usage in agriculture are outlined in Fig. 1.

Plants are continuously exposed to environmental stress. Lately, with remarkable traits of climate change, stress consequences are becoming more pronounced. Drought, overflooding, osmotic and ionic stresses caused by high salinity are some of the examples leading to considerable crop yield losses^5^. Most commonly, environmental stresses inhibit plant growth, disrupt physiological processes, lower photosynthesis, and provoke the accumulation of reactive oxygen species (ROS), leading to oxidative damage in plants. ROS accumulation in plants can trigger cellular components oxidation, loss of organelle integrity, and finally cell death^52,53^.

Multiple studies showed that polyamines spermidine and spermine, as well as putrescine, can play an important role in plant tolerance to drought^54^, salinity^55^, waterlogging^56^ or other biotic stresses^57^. Spermidine can be applied exogenously at different developmental stages to promote growth and alleviate the negative consequences of different stressors. There are numerous lines of evidence that exogenous spermidine can be applied to enhance the growth, raise endogenous polyamine levels, eliminate ROS, safeguard the activity of the antioxidant enzyme system, and preserve the integrity of plant cell function^58,59^.

Several studies investigated the exogenous spermidine on germinating soybeans and its contribution to alleviating salt stress effects. The effects were observed through the increase in sprout length, biomass, and respiration rate, increase in antioxidant enzymatic activity (superoxide dismutase, SOD; catalase, CAT; peroxidase, POD; glutathione peroxidase, GPX) and decreased malondialdehyde (MDA) and hydrogen peroxide (H_2_O_2_) contents in germinating soybeans under NaCl stress^60–62^.

Examining the polyamine behaviour in wheat seedlings under hyperosmotic stress showed that, naturally, plant response depends on its genetics, and that spermidine and spermine levels increase more in drought-tolerant genotypes than in drought-sensitive ones^63^. Similarly, it was shown that the salt-tolerant *Medicago truncatula* genotype TN1.11 had an increased polyamine/diamine (Spd^3+^ + Spm^4+^/Put^2+^) ratio compared with the salt-sensitive genotype TN6.18, suggesting a clear metabolic shift to increasing polycationic forms in response to stress^64^.

It was demonstrated that spermidine application induced salinity tolerance in wheat seedlings by enhancing photosynthetic capacity through regulation of gene expression and the activity of key CO_2_ assimilation enzymes^65^. This effect was exhibited by upregulated transcriptional levels of antioxidant enzyme genes (SOD, CAT, POD, GPX), a decrease in MDA level, an increase in chlorophyll and proline contents, and modulation of photosystem II (PSII) activity. Spermidine alleviated negative effects on CO_2_ assimilation induced by salt stress, in addition to significantly increasing the activity and content of ribulose 1,5-bisphosphate carboxylase/oxygenase (Rubisco). Similarly, it was shown that exogenous application of polyamines, including spermidine, in hydroponically grown *Citrus aurantium* seedlings under NaCl stress resulted in significant protection, linked with the induction of post-translational modifications, including the depression of protein carbonylation and tyrosine nitration, as well as the elicitation of protein *S*-nitrosylation^66^.

An emerging role of spermidine, particularly important for today’s growing demands for yield increases, can be obtained by spermidine’s spraying in the post-flowering phase. Spraying 1.5 mM spermidine for 3 days after pollination (DAP), from 3 DAP to 5 DAP, during the rice seed filling stage using 4 mL of 1.5 mM spermidine per spikelet^67^ or at 8-12 DAP, by spraying 10 mL of 0.5 mM spermidine per plant^68^ prevented the detrimental effects of later induced high-temperature stress. Spermidine helped in maintaining the 1000-grain weight, seed germination rate, germination index, vigour index, seedling establishment, and seedling characteristics during germination time. The improvement in grain filling by post-flowering application of spermidine was also observed in wheat, as it promotes the filling of inferior grains - known to require more energy to fill as a consequence of their distal or basal locations^46^ and this was also observed under drought stress conditions^69^. These positive effects were mainly achieved by affecting sucrose, starch, and amylose contents, i.e., by spermidine’s transcriptional regulation of carbohydrate metabolism-related enzymes. Exogenous spermidine also reduced the level of ethylene in the inferior grain, allowing the filling period to last longer^46,70^. In another study, the pre-soaking in spermidine of wheat grain alleviated the negative effects of irrigation with wastewater on the biochemical characteristics of the yielded grains. Indeed, this early treatment led to the formation of grain proteins of different molecular weights, thus highlighting the potential of spermidine treatment for the engineering of protein-specific grain quality in a polluted context^71^.

Similarly, in the field experiments where moderate and high wheat planting density (above 450 × 10^4^ seedlings per ha) were shown to significantly decrease the yield, 1000-grain weight, and grain number per spike, sprayed spermidine at anthesis managed to increase both starch and sucrose levels, by affecting sucrose synthase and ADP-glucose pyrophosphorylase activities, and to enhance yield^71^. Besides, spermidine application can notably promote the activities of antioxidant enzymes and decrease lipid peroxidation in the grains of plants under drought stress^69^.

The main obstacle of such treatments is the necessity of spraying for several consecutive days, which could be overcome by targeted delivery systems. Therefore, such treatments should be further optimized and proposed as a ‘green’ strategy to preserve high crop yields in the areas affected by climate change. Interestingly, optimized deliveries of spermidine and other (poly)amines could potentially be achieved through the use of advanced nanomaterials acting as smart carriers^72^, such as with efforts to deliver spermidine using silica-based nanoporous pods as an anticancer agent^73^, and with examples of agro-based approaches including the smart delivery of putrescine using carbon quantum dots against salt stress^74^ and heavy metal stress^75^ in grapevine plants.

Besides influencing macronutrient contents, as aforementioned for polysaccharides and proteins, spermidine holds the potential for the management of bioactive compounds. For example, exogenous spraying of spermidine over leaves can enhance glucosinolates in cabbage, and total phenolics in cabbage^76^ and cauliflower^77^. Logically, these treatments increase the polyamine content and antioxidant capacity of plants.

### Routes of uptake following polyamine application types in plants

Exogenously applied polyamines, whether delivered by seed soaking, soil watering or foliar spray, ultimately enter plant cells using the same transport machinery that handles endogenous polyamines. After crossing external barriers (seed coat, root surface, leaf cuticle), putrescine, spermidine, and spermine are taken up across the plasma membrane via specific polyamine uptake transporters (PUT family and related carriers), whose roles are now documented in *Arabidopsis* and crop species^78,79^. Classic tracer and compartmentation studies showed that exogenous polyamines first accumulate and bind in the apoplast, then move through a saturable, energy-dependent transport step into root and shoot cells, followed by xylem/phloem redistribution^80,81^.

The entry route and physical barrier differ with the application method. In **seed priming**, imbibition carries polyamine solutions through the seed coat into the apoplast of endosperm and embryo; as metabolism resumes, embryonic cells import polyamines via transporters, and part of this pool (and its downstream signalling changes) is retained after drying, underpinning faster germination and enhanced stress tolerance in later stages^82,83^. In **root applications**, polyamines diffuse through the soil solution into the root apoplast and are then actively taken up by epidermal and cortical cells; detailed kinetic analyses in maize roots confirm carrier-mediated uptake and apoplastic binding of exogenously supplied putrescine^80,81^. In contrast, **foliar sprays** must overcome the hydrophobic leaf cuticle; hydrophilic polyamines mainly enter through cuticular pores, cracks, and stomatal/hydathode regions, with uptake strongly modulated by droplet retention, humidity and adjuvants^84,85^. Once polyamines reach the epidermal/apoplastic space, the same PUT-type transporters and long-distance transport processes operate, so differences between spray, drench or priming are largely quantitative (dose, area exposed, timing) rather than mechanistically distinct.

### Agricultural biotechnology measures for improving spermidine content in crops - genetic and molecular engineering of polyamine metabolism

At the molecular level, the most direct strategy to increase spermidine is to enhance flux through its biosynthetic pathway. A landmark study in Arabidopsis showed that overexpression of a spermidine synthase (SPDS) gene led to markedly higher spermidine content and conferred tolerance to freezing, high salt and drought, accompanied by up-regulation of multiple stress-responsive genes^86^. Similar approaches in other species have confirmed that SPDS is a robust engineering target for boosting spermidine-linked stress resilience^87,88^.

More recent work in crops reinforces this concept. In wheat, overexpression of a spermidine synthase gene increased polyamine levels and improved resistance to Fusarium head blight without compromising yield, indicating that manipulation of spermidine metabolism can strengthen disease resistance in cereals^88^. In cotton, GhSPDS11 has been characterised as a key spermidine synthase isoform induced by alkaline stress; its overexpression is associated with elevated Spd and better physiological performance under alkaline conditions, highlighting species-specific SPDS genes as candidates for breeding and engineering programmes^89^.

Upstream enzymes that generate aminopropyl donors, such as S-adenosylmethionine decarboxylase (SAMDC) and S-adenosylmethionine synthetase (SAMS), have also been targeted. Overexpression of polyamine biosynthesis genes in model systems and crops generally increases Spd/Spermine pools and improves tolerance to salinity, drought and oxidative stress^87,90,91^. Genome-wide surveys of polyamine biosynthetic gene families now provide a toolbox of SPDS, SPMS, SAMDC and related isoforms in major crops, enabling more precise manipulation of individual family members and tissue-specific expression patterns^87^.

Newer tools such as CRISPR/Cas-based genome editing, promoter engineering and inducible expression systems offer opportunities to fine-tune spermidine accumulation in selected organs (e.g., edible tissues) or under specific stress conditions. However, polyamines are deeply involved in reproductive development, and misregulation of key biosynthetic genes can cause sterility or developmental defects^42,92^. Future agro-biotechnological approaches will therefore need to combine spatiotemporal control of gene expression with agronomic and microbial strategies to increase spermidine where and when it is most beneficial, without negative pleiotropic effects.

### Possible detrimental effects of high spermidine levels in plants

High spermidine levels can indeed have detrimental effects in plants, including the promotion of programmed cell death (PCD). Polyamines have a widely recognized dual role: they stabilise macromolecules at low concentrations, modulate signalling and protect against stress, whereas they can actively trigger or potentiate PCD at higher concentrations. Recent work in *Arabidopsis thaliana* using cell cultures and *in planta* root hair assays showed that exogenous spermidine and spermine inhibit PCD at low doses but induce PCD at higher doses in a clearly dose-dependent manner^93^. This hormetic behaviour is consistent with broader reviews concluding that polyamines can act as both pro-survival and pro-death regulators depending on their abundance, cellular compartmentation and metabolic context^94^.

## Nutritional benefits of spermidine – The role in health and disease

### Dietary sources of spermidine

Spermidine occurrence in the human body results from three sources: (i) endogenous biosynthesis, (ii) dietary intake, and (iii) gut microbiota production. Spermidine originates mainly from dietary sources^9^. Since it is a ubiquitous compound, it can be ingested by eating plant-based foods, mushrooms, or animal products. The detailed overviews of spermidine content in foods can be found in many publications^17,18,95–99^. The highest content of spermidine can be found in bean seeds, especially in soybeans, and in mushrooms, ranging approximately 100–200 mg/kg. Among animal sources, the highest quantity of spermidine is found in turban shell viscera (24–13290 mg/kg) and bovine liver (10–390 mg/kg)^17^.

**Fig. 2:**
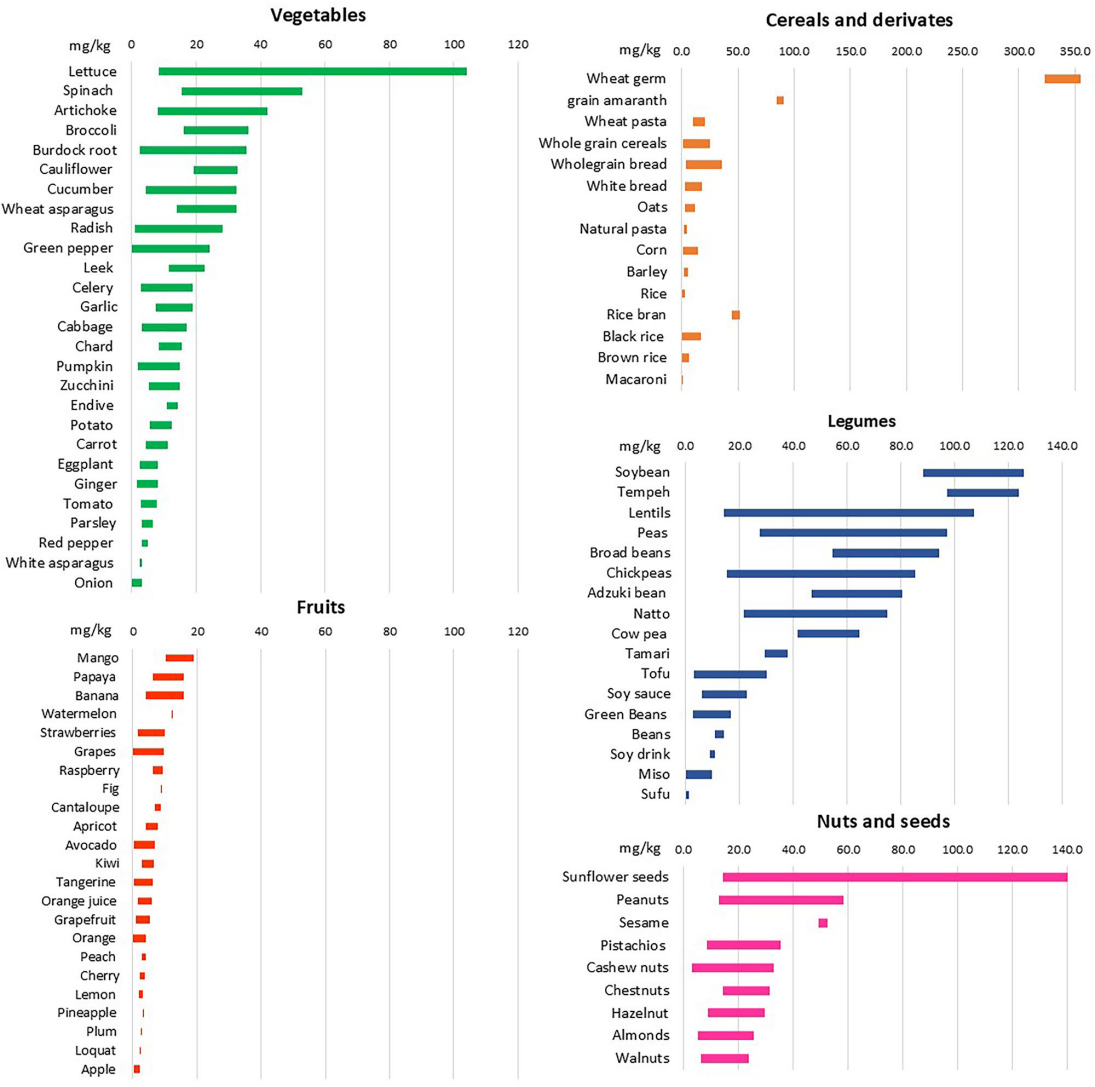
Spermidine content in plant-based foods. The content is expressed as a range in mg/kg fresh weight [the values are integrated values from refs. 95,98,99].

Spermidine content significantly varies depending on variety, plant part, and environmental factors, as shown for soybean^18,100^. In Fig. 2 the ranges of spermidine found in plant-based food are presented. Notably, even within the same food source, the content can vary dozens of times. This diversity should be taken into consideration, particularly in food production and dietary recommendations. Food processing can also significantly affect spermidine content^95^. Looking only at cereals, although wheat germs are the richest source of spermidine among plant-based foods, wheat flour and bread contain much less amount, while in macaroni and noodles, the content is almost neglectable (see Fig. 2)^98,99^. Another illustrative example goes for rice. Although rice bran can contain around 52 mg/kg of spermidine, the content in rice varies from 2 mg/kg to 17 mg/kg, depending on the variety. Therefore, rice mixed with black and brown rice, and bread mixed with wheat germ should be recommended for higher spermidine uptake^99^.

### Health effects of dietary spermidine

Regarding human health, the first association with spermidine is *healthy longevity*. The world is facing a longevity revolution. Life expectancy has risen globally for both sexes and people are living longer than ever before^101^. But the extension of lifespan is not proportionally followed by the healthspan i.e., disease-free life period^102,103^. For this reason, interest in research on spermidine is growing all over the world. Promoting autophagy, which is per se regarded as a healthy ageing mechanism, spermidine holds great potential as a geroprotective agent^16,104,105^.

Research showed that supplementation with spermidine can literally prolong the lifespan. This is well documented in flies and mice, where spermidine feeding significantly prolonged median lifespan by ~10–15%^29,106^. Importantly, this life-prolonging effect is achievable if supplementation duration is ‘life-long’ or starts ‘late in life’, which is a regimen more applicable to humans.

It is hard to measure life-prolonging effects in humans, but epidemiological data imply that nations with spermidine-rich diets live longer. Example for this are Mediterranean nations with a diet rich in beans and vegetables, or Japanese, consuming a lot of their traditional spermidine-abundant soy-based foods, such as natto, miso soup, and others^107–109^.

The data on spermidine levels in tissues and organs during the lifetime are ambiguous. Some investigations documented the decline^99,106,110^ and others showed the increase of spermidine levels during ageing^111,112^.

**Fig. 3:**
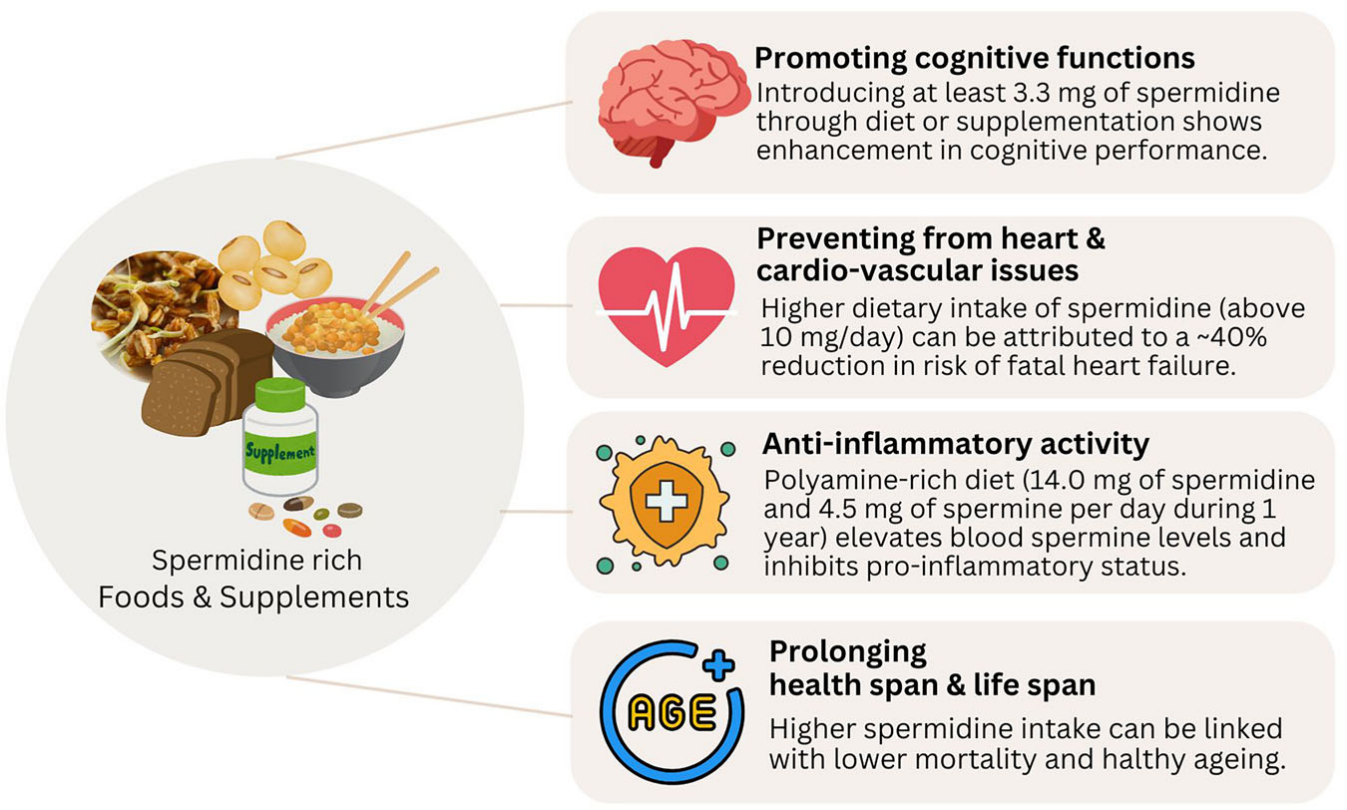
Health-promoting effects of spermidine documented in human studies. (The figure is supported by refs. 106,112,129–131 and created by Canva online tool).

There is insufficient evidence on possible ways in which spermidine demonstrates its efficacy. However, the research unambiguously shows that dietary supplementation with spermidine, whether through food rich in spermidine or as a pure compound supplementation, mediates health-beneficial effects^9,113^. Figure 3 outlines some of the health-promoting effects of spermidine documented through human studies.

It is very likely that life-prolonging effect of spermidine goes beyond a simple increase in the number of days lived, and that relies on **retardation of the manifestation of major age-associated diseases**, like cardiac and arterial aging^106,114^, colon cancer^115^, neurodegenerative processes^116,117^, and its anti-inflammatory effects^112^. For this reason, spermidine is recognised as a **healthspan prolonging** principle, being extremely important as the world population ages and chronic diseases become more prevalent. Interventions that promote **healthy aging** could serve as powerful strategies to reduce the burden of age-related diseases and their wide-ranging socioeconomic costs, providing an application potential for spermidine in the form of a pharmaceutical or nutraceutical.

The most implicated mechanism underlying spermidine’s life-prolonging effects is its ability to induce autophagy, as described earlier in Chapter 2. Spermidine acts primarily through its ability to modulate cellular acetylation status, both at nuclear and cytoplasmic (non-nuclear) effects^30^. By inhibiting key histone acetyltransferases, spermidine reduces the acetylation of histones and several autophagy-related proteins, thereby promoting chromatin states and signalling environments that favour autophagy initiation. In parallel, spermidine enhances mitochondrial function and cellular stress resistance, further supporting the autophagic response^21^.

Besides or in parallel with autophagy, several other mechanisms can be responsible or contribute to spermidine’s effects. For **brain** protection, in an animal model of Parkinson’s disease, spermidine was shown to dose dependently attenuate striatal pro-inflammatory cytokine levels (TNF-α, IL-1β, IL-6) and to restore striatal neurochemistry (the level of catecholamines: dopamine, serotonin, nor-epinephrine and their metabolites; GABA; glutamate)^117^. In **cardioprotection**, besides inducing autophagy and mitophagy in cardiomyocytes, it is shown that spermidine reduces cardiac stiffness by elevating titin phosphorylation, a key molecular process that supports the natural elasticity of cardiomyocytes and enhances the availability of arginine, the exclusive substrate for nitric oxide (NO) synthesis, leading to increased vasodilation and reduced systemic blood pressure^106^. Apart from that, improved **renal** function may additionally contribute to reduced arterial blood pressure and cardioprotection.

Across models of acute, chronic, and diabetic kidney injury, spermidine consistently exhibits **renoprotective activity** through immune modulation, metabolic regulation, antifibrotic signalling, and autophagy enhancement. In sepsis-induced acute kidney injury, spermidine improves renal outcomes by acting on macrophages, where it suppresses NLRP3 inflammasome activation and IL-1β production, preserves mitochondrial respiration, and relies on eIF5A hypusination to regulate macrophage function^118^. In chronic kidney fibrosis, spermidine derived from altered arginine metabolism activates Nrf2, thereby reducing TGF-β1 signalling, collagen expression, and mitochondrial oxidative stress; exogenous spermidine mitigates fibrosis even in Arg2-deficient mice^119^. In diabetic nephropathy, depletion of endogenous polyamines contributes to podocyte injury, whereas exogenous spermine restores podocyte integrity by activating autophagy via the AMPK–mTOR pathway, reducing apoptosis and glomerular damage^120^.

Spermidine **protects the liver** primarily by enhancing autophagy and reducing fibrogenic stress. In chronic liver injury models, dietary spermidine preserves liver endothelial cell function by lowering oxidative stress, improving mitochondrial fitness, and preventing endothelial dysfunction—an effect lost in autophagy-deficient mice, confirming its autophagy dependence. In parallel, human and mouse data show that spermidine levels decline as liver fibrosis progresses, and restoring spermidine reverses established fibrosis through the autophagy mediator MAP1S. Spermidine directly suppresses hepatic stellate cell activation, reducing ECM production and promoting lipid droplet restoration, thereby limiting fibrotic remodelling. Collectively, spermidine acts through endothelial protection, oxidative stress reduction, mitochondrial support, and MAP1S-driven autophagy to prevent or even reverse liver fibrosis^121,122^.

For the **skeletal muscle system**, it was found that spermidine intake does not affect skeletal muscle regeneration after chemical injury or hypertrophy^123,124^, but it can protect muscle or delay muscle loss, acting through autophagy mechanisms and improving mitochondrial integrity, and attenuating structural deterioration of muscle fibre^125,126^.

Spermidine and spermine can exert **bone protective effects**, where they prevent ovariectomy-induced bone loss by inhibiting osteoclast differentiation and maturation without impairing cell viability. These findings highlight that spermidine provides **robust anti-resorptive, osteoclast-targeted protection** against metabolic bone deterioration, supporting its potential use in conditions such as post-menopausal osteoporosis^127^.

Clinical studies related to dietary spermidine indicate that a long-term (for at least a year) higher intake of spermidine (above 3 mg per day) can be beneficial in multiple health-related aspects. Supplementation with 3.3 mg of spermidine via grain roll with wheat germ or introducing spermidine-rich food with at least 3.3 mg of spermidine per daily intake can promote cognitive performance, especially in people with mild dementia^128,129^. Polyamine-rich diet (~14.0 mg of spermidine and 4.5 mg of spermine per day during 1 year) elevated blood spermine levels and inhibited pro-inflammatory status in healthy male Japanese of 48.9 ± 7.9 median age^112^.

As assessed in 829 participants aged 45–84 years, from the Bruneck study run in the period 1995–2010, higher dietary intake of spermidine (above 10 mg/day) was inversely associated with risk of both fatal heart failure (~40% reduction in risk in the high- versus low-spermidine-intake groups) and clinically overt heart failure. Intake of spermidine was also inversely related to the risk of other cardiovascular diseases, as assessed by a composite of acute coronary artery disease, stroke, and death due to vascular diseases and to systolic and diastolic blood pressures^106^. The data from the same study showed the inverse relation between spermidine intake and all-cause mortality in women, men, and numerous subgroups^130^. Thus, regulatory bodies should consider nutritional strategies based on spermidine for promoting healthy aging, especially for the elderly population.

Notably, concentrations of spermidine with demonstrated health-promoting effects can be naturally found in multiple food sources, as presented in Fig. 2. However, because of the wide variation of its content within the same plant species, more attention should be paid to agricultural practices that could ensure stable spermidine content and portion-adequate levels for optimal health effects.

### Safety of spermidine dietary intake

Concerning the safety of dietary spermidine, it has a demonstrated history of safe use. An acute oral toxicity of spermidine and spermine each was determined to be 600 mg/kg body weight (bw) in Wistar rats^131^. Spermidine trihydrochloride was non-genotoxic in the in vitro assays, and no adverse effects were reported in the 90-day oral (dietary) toxicity study up to the highest dose tested, which was 728 mg/kg bw/day for males and 829 mg/kg bw/day for female rats^132^. Yet, amounts mostly consumed by diet are around 10 mg/day according to dietary spermidine intake calculated based on the Bruneck Study^130^.

Although to date no adverse effects of nutritional supply of spermidine have been reported, caution should be taken in certain health conditions such as cancer. Current evidence suggests that spermidine is generally safe and may even exert chemopreventive effects mediated by enhancing the anticancer immune response and suppressing the tumour growth^133,134^. However, because polyamines can promote cancer cell proliferation in vitro and the effects of dietary spermidine on different human cancers remain uncertain, its use in cancer patients requires caution. More research is needed before recommending spermidine-rich or polyamine-modified diets to individuals with existing malignancies^21^^,^^135^.

To the best of our knowledge, there were neither recorded nor investigated symptoms of the insufficient dietary polyamines (spermidine) intake, or recognised conditions like e.g. deficiencies observed for vitamins. Because spermidine is not only obtained from the diet but is also endogenously synthesised and produced by the gut microbiota, the total polyamine pool reflects the combined contribution of all three sources, making it difficult to detect a shortage from any single source, and therefore true dietary depletion conditions are unlikely to occur^9^.

## Pharmaceutical applications of spermidine

Spermidine possesses a potential for both systemic and local administration and effects. As an anti-inflammatory agent, spermidine was shown to suppress the levels of pro-inflammatory cytokines IL-6 and IL-1β, to increase the levels of anti-inflammatory factor IL-10, to inhibit TNF-α-induced NF-κB pathway, to reduce reactive oxygen species and lipid peroxidation. It was used to decrease liver or colonic inflammation, cartilage degeneration and osteoarthritis^17^. Topical treatment with plain spermidine and injectable spermidine hydrogel formulations reduced inflammation and enhanced wound healing^136,137^. Anti-inflammatory potential of spermidine was used for the development of injectable forms for periodontal ligament regeneration^138^. Spermidine formulations were also investigated as the scaffold for the treatment of nervous tissue injuries^139^.

Interestingly, the findings showed that spermidine improves skin health indirectly by reshaping the **skin microbiome** and restoring microbial functions that protect the epidermis. In atopic dermatitis, dysbiosis is marked by a loss of beneficial commensal metabolism, including reduced polyamine synthesis and impaired histidine–histamine conversion, which normally supports skin hydration and barrier integrity. Spermidine supplementation partly restores these microbial pathways, enhancing **epidermal lipid metabolism, ceramide-related signalling, and keratinocyte differentiation**, all of which are critical for barrier repair. Functional metagenomic analysis showed that spermidine-responsive taxa, especially *Cutibacterium acnes*, drive metabolic changes that correlate with clinical improvements, including reduced epidermal spongiosis, strengthened barrier proteins, and normalized hydration markers. Thus, spermidine acts as a **microbiome-mediated skin protectant**, promoting microbial functions that support recovery of dermal structure and barrier function^140^.

Spermidine and spermine were also found to enhance the delivery of ionic or poorly absorbed nutrients and drugs. The oral absorption efficiency of chondroitin-sulphate (a food ingredient and drug molecule for the treatment of osteoarthritis) was greatly improved by poly-ion complex formation with spermine^141^. Development of nanoparticles with poly lactide-*co*-glycolide (PLGA), polyethylene glycol (PEG), and spermidine was demonstrated as a potential strategy for targeted delivery of anticancer drugs, such as for doxorubicin and fluorofenidone^142,143^. As mentioned previously in section 2.2, exploring spermidine-based formulations, with spermidine as active agent, or part of coating material, holds great promise for future investigations and agro-food-pharma applications.

## Future perspectives and challenges

The rising global consequences of climate change, i.e., accelerated soil degradation and imposed abiotic stresses such as heat, drought, and salinity, are reducing crop productivity. When combined with an increasing human population, expected to drive up overall food demand, there is an urgent need for strategies that promote climate-resilient crops with enhanced nutritional value.

Being an integral part of the human diet and naturally found in most living organisms, spermidine is playing a vital role in essential cellular processes and shows strong potential for improving both plant and human health, particularly in enabling stress resilience. Applying spermidine to crops, also known as priming, increases plant resistance to environmental stress, boosts yields, and enriches the nutritional quality. Notably, such treatments also raise internal spermidine levels in plants, presenting a promising avenue for enhancing human dietary intake through everyday food.

Given its documented health benefits, further research is needed to fully understand spermidine’s mechanisms of action, potential side effects, and safety across different population groups. Identifying who may benefit most from dietary spermidine and where its use might need to be limited remains a key challenge for regulatory bodies.

To fully harness spermidine’s potential in building climate-resilient agriculture, improving nutrition, and supporting health, future efforts should focus on the development of green extraction techniques and targeted delivery systems. These innovations would be essential pillars of an integrated *From Farm to Pharm* strategy, bridging agriculture, food, and pharmaceutical sectors for maximum impact.

## Data Availability

No datasets were generated or analysed during the current study.
